# Supplementary material for: Cyclic-di-GMP controls Type III effector export and symptom development in Pseudomonas syringae infections via the export ATPase HrcN
Source: PLoS Pathog. 2025 Dec 26;21(12):e1013376. doi: 10.1371/journal.ppat.1013376 (PMC12774368; doi:10.1371/journal.ppat.1013376)
Supplement: S1 Table — (DOCX) [file ppat.1013376.s001.docx]

**S1 Table.** Strains and Plasmids used in this study

| **Strain Name** | **Genotype** | **Reference** |
| --- | --- | --- |
| ***Escherichia coli*** | | |
| DH5α | endA1, hsdR17(rK-mK+), supE44, recA1, gyrA (Nalr), relA1, Δ(lacIZYA-argF) U169, deoR, Φ80dlacΔ(lacZ)M15 | (1) |
| DB3.1 | gyrA462 endA1 ∆(sr1-recA) mcrB mrr hsdS20 glnV44 (=supE44) ara14 galK2 lacY1 proA2 rpsL20 xyl5 leuB6 mtl1 | Invitrogen |
| BL21-(DE3) pLysS | Sm^R^, K12 recF143 lacIq lacZΔ.M15, xylA, pLysS | Novagen |
| ***Pseudomonas syringae* pv tomato DC3000 (*Pto*)** | | |
| WT *Pto* DC3000 | Rif^R^ derivative of *P. syringae* pv tomato NCPPB 1106 | (2) |
| *Pto hrcN^G176A^* | WT *Pto* DC3000 with a G176A mutation in *hrcN* | This study |
| *Pto hrcN^E208D^* | WT *Pto* DC3000 with an E208D mutation in *hrcN* | This study |
| *Pto hrcN^G311A^* | WT *Pto* DC3000 with a G311A mutation in *hrcN* | This study |
| *Pto hrcN^L338V^* | WT *Pto* DC3000 with an L338V mutation in *hrcN* | This study |
| *Pto hrcN^F174Y^* | WT *Pto* DC3000 with an F174Y mutation in *hrcN* | This study |
| *Pto* Δ*hrcC* | WT *Pto* DC3000 with an inactivating mutation in *hrcC* | (3) |
| *Pto* Δ*hrcN* | WT *Pto* DC3000 with a non-polar deletion of *hrcN* | This study |
| *Pto* Δ*hopAA1-2* | WT *Pto* DC3000 with a non-polar deletion of *hopAA1-2* | This study |
| *Pto* Δ*hopAM1* | WT *Pto* DC3000 with a non-polar deletion of *hopAM1* | This study |
| *Pto* Δ*hopAF1* | WT *Pto* DC3000 with a non-polar deletion of *hopAF1* | This study |
| *Pto* Δ*hopAA1-2* Δ*hopAM1* | WT *Pto* DC3000 with non-polar deletions of *hopAA1-2*  & *hopAM1* | This study |
| *Pto* WT + *hrcC* | WT *Pto* DC3000 with *hrcC* inserted at the *att::*Tn*7* site. Gent^R^ | This study |
| *Pto* WT + *hrcN* | WT *Pto* DC3000 with WT *hrcN* inserted at the *att::*Tn*7* site. Gent^R^ | This study |
| *Pto* Δ*hrcC* + *hrcC* | *Pto* Δ*hrcC* with *hrcC* inserted at the *att::*Tn*7* site. Gent^R^ | This study |
| *Pto* Δ*hrcN* + *hrcN* | *Pto* Δ*hrcN* with WT *hrcN* inserted at the *att::*Tn*7* site. Gent^R^ | This study |
| *Pto hrcN^G176A^* + *hrcN* | *Pto hrcN^G176A^* with WT *hrcN* inserted at the *att::*Tn*7* site. Gent^R^ | This study |
| *Pto hrcN^E208D^* + *hrcN* | *Pto hrcN^E208D^* with WT *hrcN* inserted at the *att::*Tn*7* site. Gent^R^ | This study |
| *Pto hrcN^G311A^* + *hrcN* | *Pto hrcN^G311A^* with WT *hrcN* inserted at the *att::*Tn*7* site. Gent^R^ | This study |
| **Plasmids** | | |
| pUC18-mini-Tn*7*-Gm | Amp^R^, Gent^R^, *att::*Tn*7* insertion vector | (4) |
| pUC18-mini-Tn*7*-*hrcN/hrcC* | pUC18-miniTn*7*-GM containing WT *hrcN/hrcC* cloned between *Hind*III and *Bam*HI/*Pst*I sites | This study |
| pTNS2 | Helper plasmid for *att::*Tn*7* insertion. Amp^R^ | (4) |
| pTS1 | Tet^R^, suicide vector; *ColE1*-replicon, *IncP-1, Mob, lacZ* | (5) |
| pTS1- mutagenesis vectors | pTS1 containing various *hrcN* alleles/gene deletion constructs cloned between *Xho*I and *Bam*HI sites | This study |
| pBBR1MCS-4 | Broad host-range expression vector. MCS with *lacZ* blue/white selection and upstream *lac* promoter. Amp^R^ | (6) |
| pBBR4-*bifA* | pBBR1MCS-4 expressing *Pseudomonas fluorescens* SBW25 *bifA* | This study |
| pBBR4-*wspr19* | pBBR1MCS-4 expressing the *P. fluorescens* SBW25 *wspR19* allele | This study |
| pETM11 | Protein expression vector, enabling N-terminal His_6_ tag fusions. Kan^R^ | (7) |
| pETM11-*hrcN* vectors | pETM11 with *hrcN* alleles cloned between *Nde*I *and* *Xho*I sites | This study |
| pBBR1MCS-2 | Broad host-range expression vector. MCS with *lacZ* blue/white selection and upstream *lac* promoter. Kan^R^ | (6) |
| pBBR2-*hopAA1-2* | pBBR1MCS-2 with *hopAA1-2* cloned between *Kpn*I *and* *Xho*I sites | This study |
| pBBR2*-hopAM1* | pBBR1MCS-2 with *hopAM1* cloned between *Kpn*I *and* *Xho*I sites | This study |
| pBBR2*-hopAF1* | pBBR1MCS-2 with *hopAF1* cloned between *Kpn*I *and* *Xho*I sites | This study |
| pBBR2*-hopH1* | pBBR1MCS-2 with *hopH1* cloned between *Kpn*I *and* *Xho*I sites | This study |
| pCPP5371 | Gateway destination vector for expression of *Pto* effectors from the *avrPto* promoter with C-terminal *cya* tags. Gent^R^, Cm^R^ | (8) |
| pENTR-SD/D-TOPO vectors | pENTR-SD/D-TOPO gateway cloning vectors containing *Pto* effector genes lacking stop codons. Kan^R^ | (9) |
| pDEST vectors | pCPP5371 vectors containing *Pto* effector genes with C-terminal *cya* tags. Gent^R^, Cm^R^ | This study |
