# Supplementary material for: Cyclic-di-GMP controls Type III effector export and symptom development in Pseudomonas syringae infections via the export ATPase HrcN
Source: PLoS Pathog. 2025 Dec 26;21(12):e1013376. doi: 10.1371/journal.ppat.1013376 (PMC12774368; doi:10.1371/journal.ppat.1013376)
Supplement: S2 Table — (DOCX) [file ppat.1013376.s002.docx]

**S2 Table.** Primers used in this study

| **Oligonucleotide** | **Sequence** |
| --- | --- |
| DC3000-1-HrcN-FWD | CCGCTCGAGCCAGGAATGCACGCTGC |
| DC3000-1-HrcN-REV | GCGGATCCCAGCGGTTCGCCCGATG |
| DC3000-2-HrcN-FWD | CCGCTCGAGACGTCATCGTCTTCGGG |
| DC3000-2-HrcN-REV | GGTACTCGAGCGTTCAGTTGCACCG |
| E208D-FWD-DC3000-HrcN | GGCCGCGACCTGCGCGA |
| E208D-REV-DC3000-HrcN | CGCGCAGGTCGCGGCCC |
| G311A-FWD-DC3000-HrcN | TGAGCGAAAACGCTTCGATCACCG |
| G311A-REV-DC3000-HrcN | CGGTGATCGAAGCGTTTTCGCTCA |
| L338V-FWD-DC3000-HrcN | GCTCGTTGGTCGACGGCCA |
| L338V-REV-DC3000-HrcN | TGGCCGTCGACCAACGAGC |
| FWDExternalHrcN | CAGCAGGACCTGGCGCTG |
| REVExternalHrcN | TCGCGGCGGGCAAAGCC |
| OE_DC3000_FWD | CAGAAGCCATATGGTGAACGCCGCACTGAAC |
| OE_DC3000_REV | GGTACTCGAGTTACTCCGGCAGTTGCGA |
| F174H FWD (HrcN) | CGCTGCAGGACACCCACG |
| F174H REV (HrcN) | CGCATATGGAGCAACGAGCGTACT |
| FWD Cya 'P3' Reporter Plasmid Primer | TGAGCATGCTACCGAGTAACGCAGCT |
| REV Cya 'P4' Reporter Plasmid Primer | AGTGGTACCGATATCGAATTCTTAGCTGT |
| HrcNCompFWD | GTCAAGCTTGCGTTTCAAGGACC |
| HrcNCompREVPstl | GACACTAGTTTACTCCGGCAGTT |
| HrcCCompFWD | GCTAAGCTTCCATCGATCCGCAG |
| HrcCCompREV | ATCGGATCCTCATGGTTTCGCTC |
| pTN7R | CACAGCATAACTGGACTGATTTC |
| pGlmS-Down | GCACATCGGCGACGTGCTCTC |
| OvExp HopAM1 FWD | GAAGCGGTACCATGCACGCAAATCCT |
| OvExp HopAM1 REV | TGGTACTCGAGTTAGTCGCCTAGGAA |
| Del HopAM1 OutFWD | CGCATATGGGTATCGATGATGCC |
| Del HopAM1 InnREV | CGTCTAGAAGGATTTGCGTGCAT |
| Del HopAM1 InnFWD | CGCTCTAGATTCCTAGGCGACTAA |
| Del HopAM1 OutREV | CGGGATCCGTCGCTAATGGAGCT |
| OvExp HopAA12 FWD | GAAGCGGTACCATGCACATCAACCAA |
| OvExp HopAA12 REV | TGGTACTCGAGTTACAAACGCCTGAG |
| Del HopAA12 OutFWD | CTCATATGACCCACGCTTTTGCG |
| Del HopAA12 InnREV | CGCTCTAGAGGAAATTCTATCTCG |
| Del HopAA12 InnFWD | CGTCTAGAGCGGCCTGTGGGTTG |
| Del HopAA12 OutREV | CGGGATCCTGTGGTGTGGTGTCG |
| OvExp HopAF1 FWD | GAAGCGGTACCATGGGGCTATGTATT |
| OvExp HopAF1 REV | TGGTACTCGAGTTATTGTGCGACCAG |
| Del HopAF1 OutFWD | CTCATATGTGCAGTATGTAGGCT |
| Del HopAF1 InnREV | CGCTCTAGAAATACATAGCCCCAT |
| Del HopAF1 InnFWD | CGCTCTAGACTGGTCGCACAATAA |
| Del HopAF1 OutREV | ATGGATCCGCGCTGAAAACGCAA |
| AF1DelOutFWD V2 PstI` | CGCTGCAGTGCAGTATGTAGGCT |
| Q1DelOutFWD V2 PstI | CGCTGCAGATCAGCGCAATTTTC |
| HopH1 OvExp FWD KpnI | GAAGCGGTACCATGATCACTCCGTCT |
| HopH1 OvExp REV XhoI | TGGTACTCGAGCTATTGATGTGCCCT |
| HopH1 del OutFWD NdeI | CTCATATGTTTGCGCATCTGCGC |
| HopH1 del InnREV XbaI | CGTCTAGAAGACGGAGTGATCAT |
| HopH1 del InnFWD XbaI | CGCTCTAGAAGGGCACATCAATAG |
| HopH1 del OutREV BamHI | CTGGATCCACCAAGCTGGCG |

1. D. M. Woodcock *et al.*, Quantitative evaluation of Escherichia coli host strains for tolerance to cytosine methylation in plasmid and phage recombinants. *Nucleic Acids Res* **17**, 3469-3478 (1989).

2. D. A. Cuppels, Generation and Characterization of Tn5 Insertion Mutations in Pseudomonas syringae pv. tomato. *Appl Environ Microbiol* **51**, 323-327 (1986).

3. M. G. Kim *et al.*, Two Pseudomonas syringae type III effectors inhibit RIN4-regulated basal defense in Arabidopsis. *Cell* **121**, 749-759 (2005).

4. K. H. Choi *et al.*, A Tn7-based broad-range bacterial cloning and expression system. *Nature methods* **2**, 443-448 (2005).

5. T. A. Scott, D. Heine, Z. Qin, B. Wilkinson, An L-threonine transaldolase is required for L-threo-beta-hydroxy-alpha-amino acid assembly during obafluorin biosynthesis. *Nature Communications* **8** (2017).

6. M. E. Kovach *et al.*, Four new derivatives of the broad-host-range cloning vector pBBR1MCS, carrying different antibiotic-resistance cassettes. *Gene* **166**, 175-176 (1995).

7. A. Dümmler, A. M. Lawrence, A. de Marco, Simplified screening for the detection of soluble fusion constructs expressed in *E. coli* using a modular set of vectors. *Microb Cell Fact* **4**, 34 (2005).

8. H. S. Oh, B. H. Kvitko, J. E. Morello, A. Collmer, *Pseudomonas syringae* lytic transglycosylases coregulated with the type III secretion system contribute to the translocation of effector proteins into plant cells. *J Bacteriol* **189**, 8277-8289 (2007).

9. L. M. Schechter, K. A. Roberts, Y. Jamir, J. R. Alfano, A. Collmer, Pseudomonas syringae type III secretion system targeting signals and novel effectors studied with a Cya translocation reporter. *J Bacteriol* **186**, 543-555 (2004).
