# Supplementary figures and images for: Cyclic-di-GMP controls Type III effector export and symptom development in Pseudomonas syringae infections via the export ATPase HrcN
Source: PLoS Pathog. 2025 Dec 26;21(12):e1013376. doi: 10.1371/journal.ppat.1013376 (PMC12774368; doi:10.1371/journal.ppat.1013376)

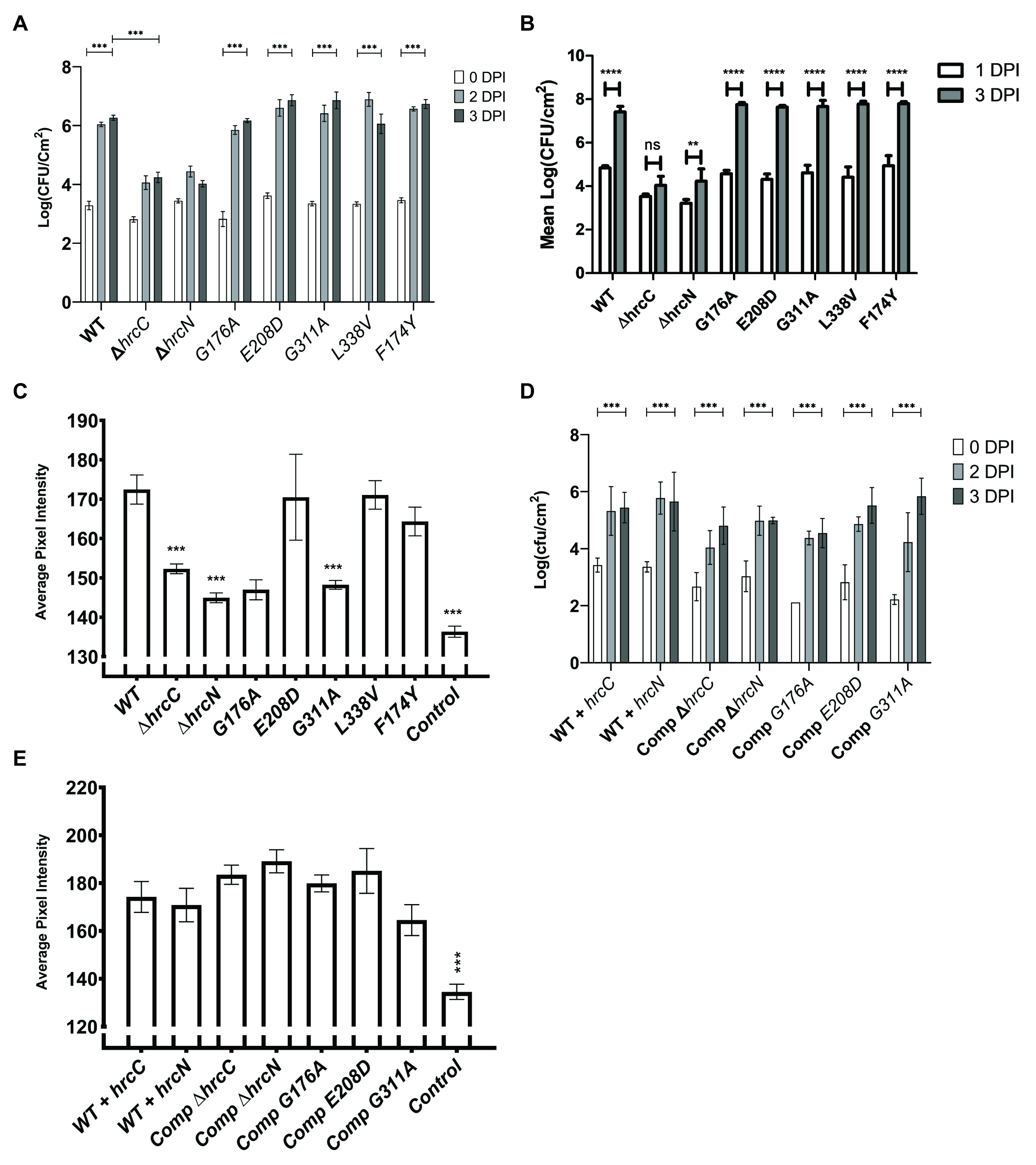

Supplement: S1 Fig — A. Colony forming units (CFU) recovered from A. thaliana Col-0 leaves infiltrated with Pto DC3000 hrcC/hrcN mutants at 0, 2 and 3 days post-infection (DPI) as indicated. B. CFU recovered from A. thaliana Col-0 leaves infiltrated with Pto DC3000 hrcC/hrcN mutants after 1 and 3 DPI. 3DPI samples were used for anti-HrcN Western blotting in Fig 1D. C. Average pixel intensity analysis for A. thaliana Col-0 leaves infiltrated with Pto DC3000 hrcC/hrcN mutants 6 days post-infection. Analysis was performed using ImageJ software (version 1.52a) and increased intensity is directly proportional to the extent of leaf yellowing. Control indicates uninfected leaf tissue. D. CFU recovered from A. thaliana Col-0 leaves infiltrated with Pto DC3000 hrcC/hrcN complementation strains at 0, 2 and 3 DPI as indicated. E. Average pixel intensity analysis for A. thaliana Col-0 leaves infiltrated with Pto DC3000 complementation strains 6 days post-infection. Analysis was performed using ImageJ software (version 1.52a) and increased intensity is directly proportional to the extent of leaf yellowing. Control indicates uninfected leaf tissue. In each case, different hrcC/hrcN alleles are indicated on the X-axis. Error bars show standard error of the mean, and asterisks denote statistically significant differences from the WT/D0 (2 sample t-test) where ‘**’ denotes p = ≤ 0.01, ‘***’ (‘****’ in B) denotes p = ≤ 0.001. A: n = 4 plants; B, D: n = 3 plants; C,E: n = 8 leaves. (TIF) [file ppat.1013376.s003.tif]

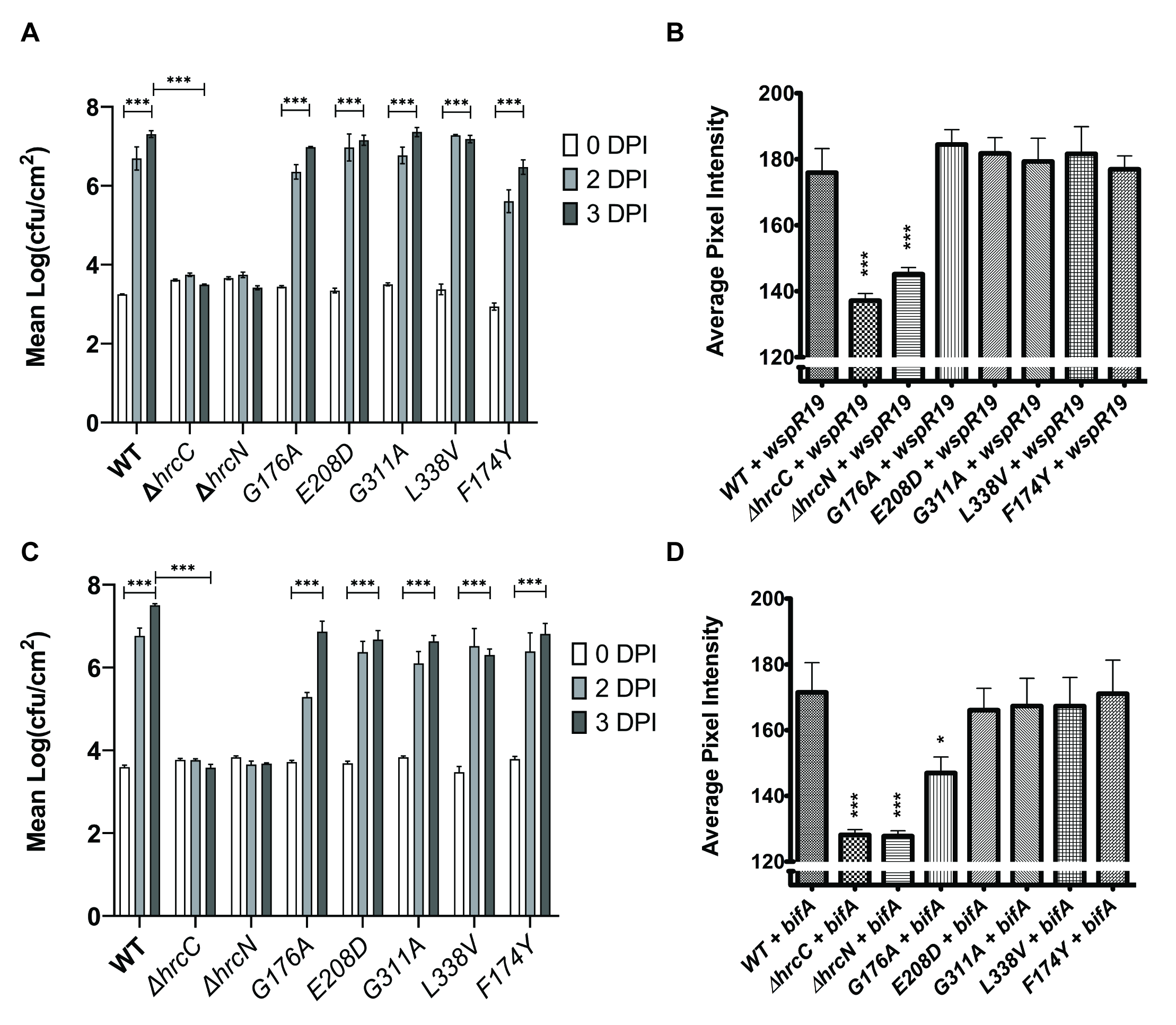

Supplement: S2 Fig — A. CFU recovered from A. thaliana Col-0 leaves infiltrated with Pto DC3000 wspR19-expressing strains at 0, 2 and 3 DPI as indicated. B. Average pixel intensity analysis for A. thaliana Col-0 leaves infiltrated with Pto DC3000 wspR19-expressing strains 6 days post-infection. Analysis was performed using ImageJ software (version 1.52a) and increased intensity is directly proportional to the extent of leaf yellowing. C. CFU recovered from A. thaliana Col-0 leaves infiltrated with Pto DC3000 bifA-expressing strains at 0, 2 and 3 DPI as indicated. D. Average pixel intensity analysis for A. thaliana Col-0 leaves infiltrated with Pto DC3000 bifA-expressing strains 6 days post-infection. Analysis was performed using ImageJ software (version 1.52a) and increased intensity is directly proportional to the extent of leaf yellowing. In each case, different hrcC/hrcN alleles are indicated on the X-axis. Error bars show standard error of the mean, and asterisks denote statistically significant differences from the WT/D0 (2 sample t-test) where ‘*’ = p ≤ 0.05 and ‘***’ = p ≤ 0.001. A,C: n = 3 plants; B,D: n = 8 leaves. (TIF) [file ppat.1013376.s004.tif]

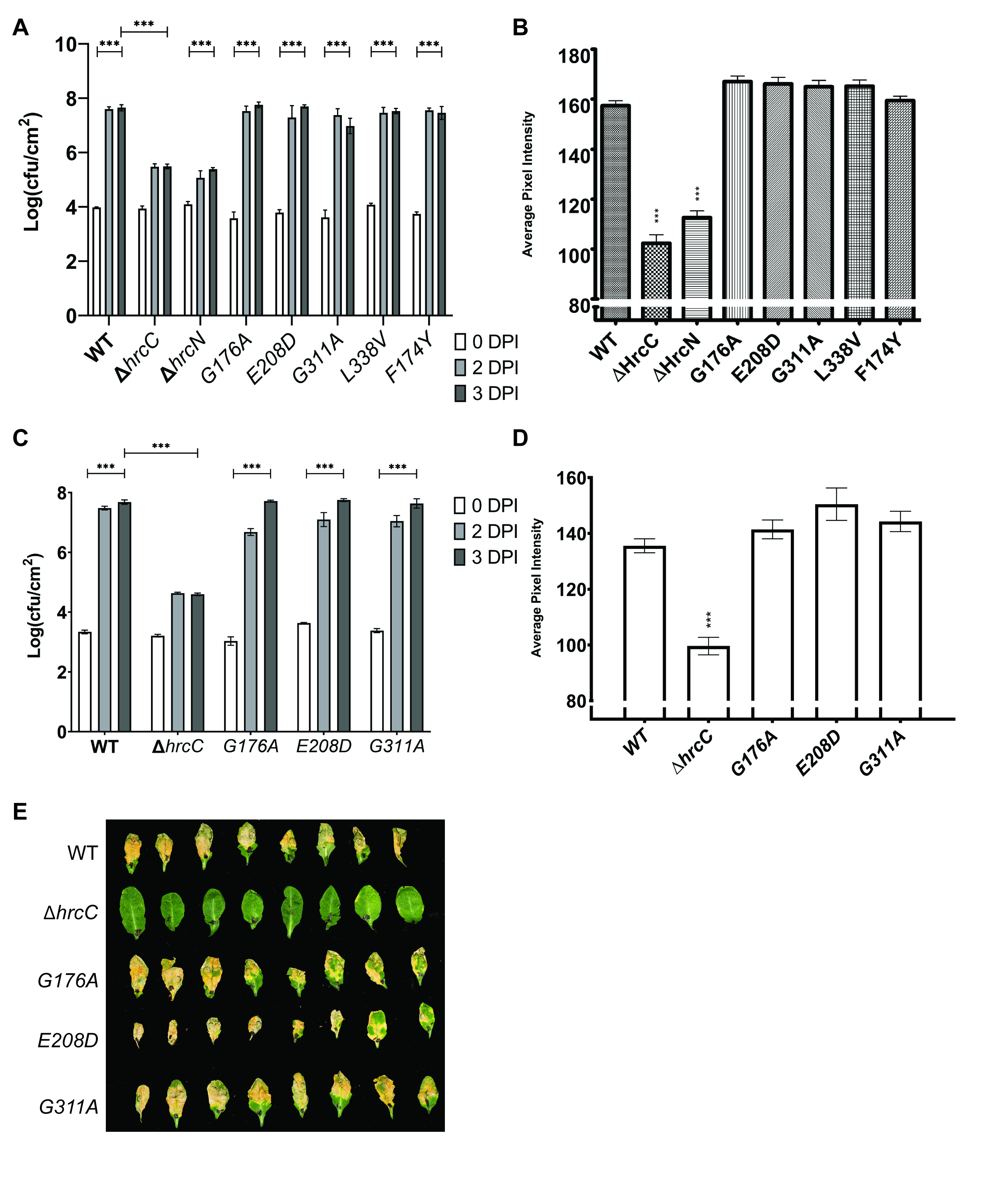

Supplement: S3 Fig — A. CFU recovered from A. thaliana bbc (immunocompromised) leaves infiltrated with Pto DC3000 hrcC/hrcN mutants at 0, 2 and 3 DPI as indicated. B. Average pixel intensity analysis for A. thaliana bbc leaves infiltrated with Pto DC3000 hrcC/hrcN mutants 6 days post-infection. Analysis was performed using ImageJ software (version 1.52a) and increased intensity is directly proportional to the extent of leaf yellowing. C. CFU recovered from A. thaliana fec leaves infiltrated with Pto DC3000 hrcC/hrcN mutants at 0, 2 and 3 DPI as indicated. D. Average pixel intensity analysis for A. thaliana fec (immunocompromised) leaves infiltrated with Pto DC3000 hrcC/hrcN mutants 6 days post-infection. Analysis was performed using ImageJ software (version 1.52a) and increased intensity is directly proportional to the extent of leaf yellowing. In each case, different hrcC/hrcN alleles are indicated on the X-axis. Error bars show standard error of the mean, and asterisks denote statistically significant differences from the WT (2 sample t-test) where ‘***’ denotes p = ≤ 0.001. A,C: n = 3 plants; B,D: n = 8 leaves. E. Visual disease phenotypes of Pto DC3000 infiltrated A. thaliana fec leaves taken at random from 3 independent plants, 6 days post-infection. Pto strains harbour the hrcC/hrcN alleles as indicated. (TIF) [file ppat.1013376.s005.tif]

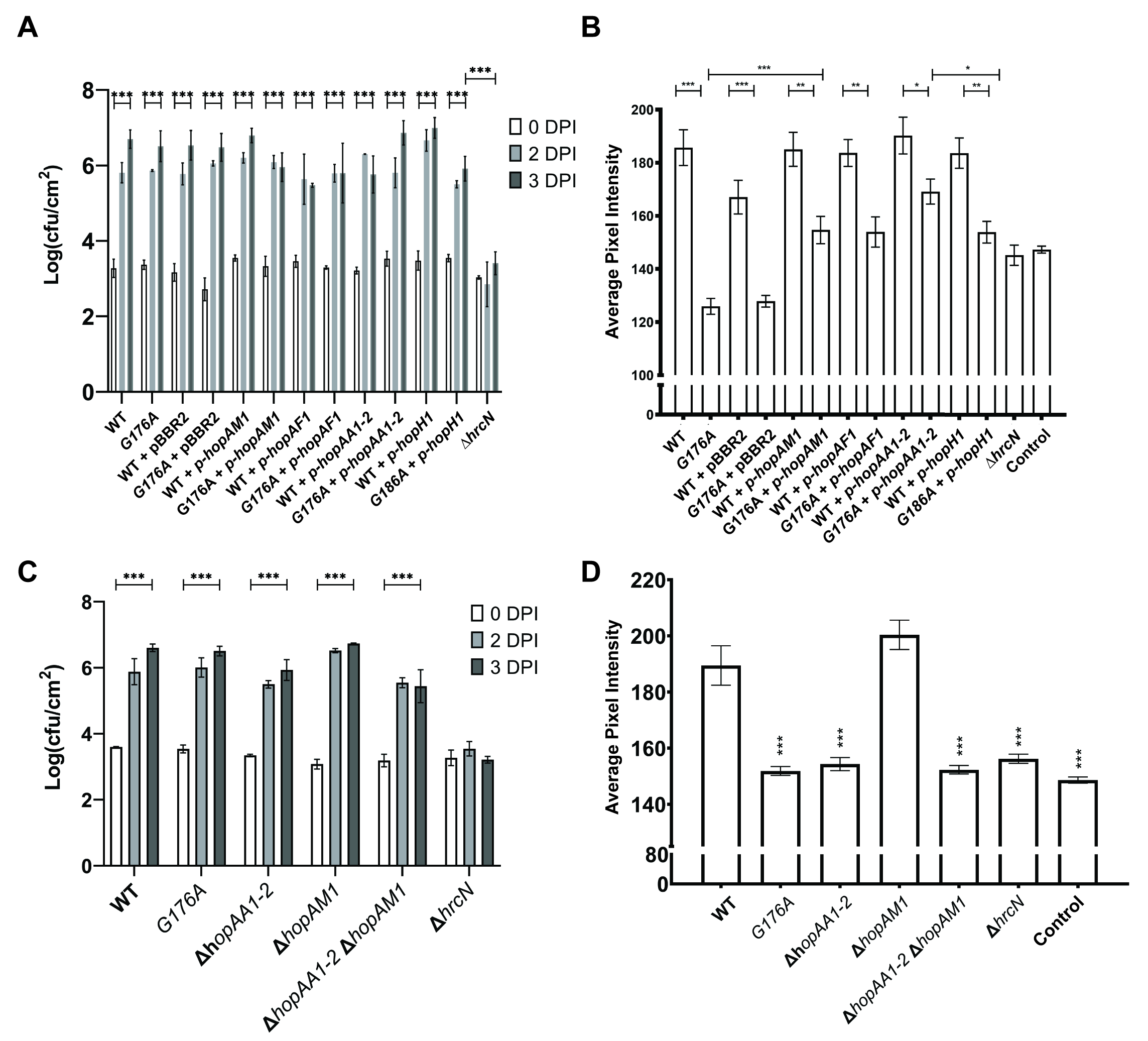

Supplement: S4 Fig — A. CFU recovered from A. thaliana Col-0 leaves infiltrated with Pto DC3000 effector overexpression strains at 0, 2 and 3 DPI as indicated. B. Average pixel intensity analysis for A. thaliana Col-0 leaves infiltrated with Pto DC3000 effector overexpression strains 6 days post-infection. Analysis was performed using ImageJ software (version 1.52a) and increased intensity is directly proportional to the extent of leaf yellowing. Control indicates uninfected leaf tissue. C. CFU recovered from A. thaliana Col-0 leaves infiltrated with Pto DC3000 effector deletion mutants at 0, 2 and 3 DPI as indicated. D. Average pixel intensity analysis for A. thaliana Col-0 leaves infiltrated with Pto DC3000 effector deletion mutants 6 days post-infection. Analysis was performed using ImageJ software (version 1.52a) and increased intensity is directly proportional to the extent of leaf yellowing. Control indicates uninfected leaf tissue. In each case, different hrcN/effector mutants are indicated on the X-axis. Error bars show standard error of the mean, and asterisks denote statistically significant differences from the WT/D0 (2 sample t-test) where ‘*’ = p ≤ 0.05, ‘**’ = p ≤ 0.01, and ‘***’ = p ≤ 0.001. A,C: n = 3 plants; B,D: n = 8 leaves. (TIF) [file ppat.1013376.s006.tif]
