# Supplementary material for: Cyclic-di-GMP controls Type III effector export and symptom development in Pseudomonas syringae infections via the export ATPase HrcN
Source: PLoS Pathog. 2025 Dec 26;21(12):e1013376. doi: 10.1371/journal.ppat.1013376 (PMC12774368; doi:10.1371/journal.ppat.1013376)
Supplement: Attachment — Submitted filename: 20250902 Rebuttal.docx [file ppat.1013376.s009.docx]

**Cyclic-di-GMP controls Type III effector export and symptom development in *Pseudomonas syringae* infections via the export ATPase HrcN: Response to Reviewers**

We thank the reviewers for taking the time to give valuable feedback on our manuscript. We have revised the manuscript throughout to address their concerns and are confident that this has resulted in a significantly stronger final study. Responses to individual points are listed below.

**Part I – Summary**

Please use this section to discuss strengths/weaknesses of study, novelty/significance, general execution and scholarship.

**Reviewer #1:** This study investigates how the bacterial second messenger cyclic-di-GMP (cdG) regulates the Type III secretion system (T3SS) in *Pseudomonas syringae*, with a particular focus on the ATPase HrcN. Mutations in two residues (G176A and G311A), predicted to interact with cdG, result in attenuated plant disease symptoms while leaving bacterial proliferation in *Arabidopsis* intact. The authors convincingly decouple symptom development from *in planta* growth and link the phenotype to altered secretion of a subset of effectors, particularly HopAA1-2, which appears essential for symptom development. The manuscript is well written, and the experimental framework is robust, combining genetic, biochemical, and plant infection assays. The use of effector-CyaA translocation assays and immune-compromised plant lines adds further depth. The study provides interesting insight into how second messengers such as cdG can post-translationally modulate T3SS function, an area of growing interest in bacterial pathogenesis.

However, the mechanistic interpretation remains somewhat speculative due to some limitations: the biochemical characterization of only one mutant (G176A), despite G311A showing an equally pronounced phenotype; and the observation that G176A still binds cdG despite its phenotype, which complicates the model that cdG binding per se is required for regulatory control. Additional experiments are needed to resolve whether mutations disrupt cdG binding affinity, downstream allosteric signaling, or HrcN complex stability. The study is timely and significant, but clarification of these mechanistic issues is important.

**Author Response:** We thank Reviewer #1 for their positive comments on our manuscript. As outlined in the “Major Issues” section below, we have revised our description of the biochemical analysis of HrcN and highlighted the outstanding uncertainties concerning the mechanisms of HrcN-cdG interaction.

**Reviewer #2:** This work is based on previous results from the team showing that cdGMP binds to a type of ATPases which includes T3SS ATPase from *Pseudomonas syringae* HrcN. Following the rationale that binding to the ATPase needed for such a key virulence determinant to work is expected to have biological consequences which have not been previously explored. Based on AlphaFold predictions, several residues predicted to be involved in cdGMP binding were selected for mutagenesis and characterization. These mutations do not alter bacterial colonization of plant tissues, but some affect the appearance of associated disease symptoms leading to the authors proposing that their impact may be related to translocation of some but not all effectors, and their testing this by following translocation of 23 effectors.

The initial premise of this report is an interesting one since T3SS ATPases are indeed important for a critical virulence determinant and very little has been done to characterize their biological roles in plant pathogens. In fact, one issue that should be addressed by the authors is that in its current form, although the vast majority of the relevant knowledge comes from mechanistic and structural insight obtained from a few animal pathogens, this is often not clear in the text. Although it is correct to take that as working hypothesis for other T3SS, it is important to make clear that it has only been shown for that T3SS. Similarly, effector translocation is presented as coordinated to follow a time hierarchy, but the only reference included is for *Salmonella* (ref 38 line 65). Specific mentions to plants do link different effectors to differential impacts on early or late infection events, however, although early events must imply early translocation, phenotypes associated to late events do not necessarily mean late effector translocation since the impact for an early molecular interaction may be only noticeable later on. Additionally, when this issue is presented again in the discussion section (144-145), it is again presented as a generally established fact, this time including two references, ref 38 from Salmonella as before, and a plant-linked reference (6) where this issue is mentioned as a future challenge not as a proven fact. These generalizations often lead to assumptions on aspects that have not been properly proven, and in this particular case, does not help to properly frame the relevance of their work, which is enhanced by the scarcity of reports on the mechanistic characterization of these systems beyond a few animal pathogens. Thus, the authors should thoroughly review the introduction and discussion to carefully address citations to avoid this type of issues.

Additionally interesting aspects of this report include the infrequent separation of impacts on symptom induction and bacterial proliferation found for mutations in HrcN residues selected on the basis of their potential relevance for cdGMP binding. Although common to other types of plant pathogens (e.g. viruses) most mutations affecting disease development in plant pathogenic bacteria affect bacterial colonization. The link to differential effector translocation, and in particular to the translocation of specific effectors that affect induction of symptoms without affecting bacterial colonization is another interesting novelty of the report. Altogether, the research presented is of wide interest to the field.

**Author Response:** We thank Reviewer #2 for their detailed and insightful feedback on the manuscript. We have rewritten several sections of the introduction and discussion (e.g. lines 50-59, 65-72, 82-84 and 220-224) to scale back some of our more universal statements concerning T3I function and to clarify the microbes examined in each cited study.

**Reviewer #3:** In this study, Ward and colleagues investigate how a putative interaction between cyclic diguanylate (c-di-GMP) and the HrcN ATPase. HrcN is a protein that interacts with the type 3 injectisome (T3I) – a molecular syringe that delivers toxic effectors to target host cells. The T3I is important for the virulence of many *Pseudomonas* species. This includes the plant pathogen *Pseudomonas syringae* that is the focus of this study.

The context of the research is well positioned relative to research with other *Pseudomonas* species and the interaction of c-di-GMP with ATPases in other systems. How c-di-GMP affect type 3 secretions systems is an open question in the field. The current study addresses that knowledge gap.

Through a series of genetic, biochemical and functional assays in *Arabidopsis thaliana*, the authors seek to demonstrate that c-di-GMP binding to HrcN adjusts the range of effector proteins secreted by the T3I, thereby influencing the outcome of plant infections. The results, if accurate, are an advance to fundamental knowledge of signal transduction that will be of interest to many in the field. However, I have a few concerns with the interpretation of data, and I’m not convinced that all the conclusions are justified. I hope the authors will consider and address this feedback.

**Author Response:** We thank Reviewer #3 for their detailed and positive comments on our work. We acknowledge their concerns with the current draft. In response we have conducted additional biochemical assays and extensively revised the manuscript, both to clarify the text and strengthen our conclusions.

**Part II – Major Issues: Key Experiments Required for Acceptance**

*Please use this section to detail the key new experiments or modifications of existing experiments that should be absolutely required to validate study conclusions. Generally, there should be no more than 3 such required experiments or major modifications for a "Major Revision" recommendation. If more than 3 experiments are necessary to validate the study conclusions, then you are encouraged to recommend "Reject".*

**Reviewer #1:** The DRaCALA binding assay results (Fig. 2B–C) suggest that both WT and G176A bind cdG, but the competition assays imply altered affinity in G176A. However, this is not quantified. ITC or other biochemical experiments is needed (or at least densitometric quantification of DRaCALA) to compare cdG binding affinities of WT and G176A. This is important to support one of the proposed mechanism that mutations modulate effector export via altered cdG-HrcN interactions.

Only G176A was characterized biochemically. G311A, which shows a similar infection phenotype, was not analyzed due to solubility issues. However, excluding it weakens the generalizability of the conclusions. In addition, it would be interesting to test additional mutants (e.g., E208D, L338V, F174Y) for cdG binding and ATPase activity to validate whether phenotypically neutral mutations retain biochemical function. Alternatively, attempt solubilization of G311A using different buffers, expression systems, or tags.

The authors show that only a subset of effectors are affected in G176A/G311A, but it is unclear whether this reflects post-transcriptional effects, altered recognition, or secretion docking.

**Author Response:** We thank Reviewer #1 for their suggestions here. We agree that additional biochemistry would strengthen our mechanistic understanding and conducted a series of extra experiments with the G176A and G311A mutants to attempt to address this. Unfortunately, and despite considerable effort, we were unable to advance our biochemical analysis as much as we would have hoped. The G311A variant is both poorly expressed and slightly insoluble in every buffer we tried. We were able to confirm ATPase activity for this mutant, now included in Fig 2, and obtained some hints that cdG still binds to it.

**Figure R1:** SPR sensorgrams show concentration-dependent binding of cdG to HrcN variants.

We also attempted several other biophysical assays with HrcN, of which SPR was the most successful (see Figure R1), with concentration-dependent binding seen for all three tested HrcN variants. However, the binding responses from these assays are pretty low, and we are reluctant to include this rather weak data in our study.

We have updated Fig 2 and the associated text (lines 148-163) to qualify our claims regarding the biochemistry of the cdG-HrcN interaction. We have also expanded our discussion to clarify the remaining uncertainty about the possible mechanisms for cdG control of HrcN function and effector secretion (lines 243-254).

**Reviewer #2:** -Mutant variants of HrcN are tested by Western blot for impact on T3SS assembly using antibodies to detect HrcN and HrcQ. However, no control for loading (normalization of the samples) is mentioned in either the text, the methods or the fig. legend. Since differences in band intensity are visible in the HrcN and HrcQ (in HrcQ and contaminating FliI) membranes, and a quantitative difference could be potentially relevant, such internal control and relative quantification is important.

**Author Response:** Our aim with the Western detection in Fig 1D was simply to show that HrcN was absent in the ∆*hrcC* and ∆*hrcN* samples but was still present in the two asymptomatic *hrcN* mutants (G176A and G311A). Therefore, we did not initially consider quantification when making our original figures. Nonetheless, the reviewer makes a good point here, which hopefully we are well placed to address. The Western blotting in Fig 1D was a spin-off experiment from a repeat of the plant infection assay in 1C. As such, samples of each leaf tissue lysate were plated and CFUs counted prior to SDS buffer addition and Western blotting, providing a robust quantification of bacterial load going into each sample. We have included these data as a new supplementary panel S1B Fig. We see no significant difference in CFU/cm^2^ leaf tissue between any of our *hrcN* mutants or WT *Pto*. ∆*hrcC* and ∆*hrcN* samples show lower CFUs/cm^2^, as expected.

Unfortunately, we were unable to provide similar quantification for the HrpQ Western. As it is somewhat tangential to the story, we decided to remove this dataset from the manuscript to maintain consistency.

-In addition, the fact that the proteins are detected in the infected-leaf samples does not mean that the T3SS apparatus is correctly assembled (lines 116-119), since the authors are not even testing subcellular locations (i.e. membrane bound fractions) but whole extracts, so their conclusions (once ratified by proper quantification) can include the absence of changes in protein production, not assembly. Results and discussion sections should be reviewed in relation to this issue.

**Author Response:** We have amended the discussion section (lines 228-233) to clarify that HrcN detection does not guarantee that a correctly assembled T3I is present. Nonetheless, our results together strongly argue for the presence of a fully assembled, if compromised, T3I in G176A and G311A. For example, 1) the *G176A* and *G311A* mutants proliferate normally in plant infection assays, unlike ∆*hrcN/*∆*hrcC* (Figure S1). It is well established that a functional T3I is necessary for *Pto* to colonise and proliferate in plant tissue (Collmer et al., 2000); 2) *G176A* and *G311A* mutant infections of immunocompromised *bbc* plants are indistinguishable from WT, again unlike ∆*hrcN* (Figure 3); and 3) secretion of multiple effectors, e.g. HopH1 is unaffected in either mutant background (Figure 4).

Collmer et al., 2000 – DOI: 10.1073/pnas.97.16.8770

-Also related to western blot results, the figure legend includes mention to FliI being detected as a contamination and the identity of all three proteins (HrcN, HrcQ and FliI) being established by mass spectrometry. However, no additional mention to this is found neither on the result nor the methods section. This should be included at least in the corresponding Western blot analysis section of the methods.

**Author Response:** This was an oversight, and we thank the reviewer for bringing it to our attention. We have updated the methods section to include details of the Mass Spec. verification of protein targets (line 418-421).

-In figures where images are shown to illustrate impact on symptom induction, figure legends include mention to how many leaves and plants are shown, which are described as representative, but lack reference to the final N analyzed. The method section only describes Image J analysis, and there the minimum N used is mentioned, but it is inconsistent at least in some cases with the numbers included in the figure legend. For example, Fig.1C is said to show 6 representative leaves from 4 plants, while the image J methods describe a minimum of 8 leaves per experiment. Are these 6 leaves representative of 8 leaves? Then why not show all? If representative of more, it should indicate so in the specific legend. Also, should indicate if 4 plants from the same or independent experiments. These issues should be fixed by including the specific N or each of these experiments in the specific legends for all figures showing this type of data.

**Author Response:** The discrepancy here arose during figure preparation. While 8 leaves were imaged and analysed in each experiment, when designing our figures we only showed six of these for aesthetic reasons. We apologise for any confusion this may have caused and have remade our figures throughout to include all eight leaves analysed. We have also revised the figure legends to clarify N values and the experimental design in each case.

-The results showing that mutations to the residues affecting symptom induction still bind cdGMP are puzzling. Since these mutant variants display altered dynamics, and the link to cdGMP is well established by overexpression of WspR, the relation between the phenotypes and cdGMP regulation appears to be there. However, this issue should be covered in the discussion as it is an important mechanistic aspect of the work that remains to be established. In relation to this, the results obtained after overexpression of BifA is also another puzzling result that should be properly discussed.

**Author Response:** The relationship between cdG production, HrcN function and plant infection is highly complex, and obtaining clear, unambiguous results from overexpression experiments is difficult. We have expanded the discussion as requested (lines 255-263) to more thoroughly account for the discrepancies we see in our mutant/cdG manipulation assays.

**Reviewer #3:** Lines 92-101 and Fig. 1. Could the authors provide more information about the validation of AlphaFold3 structures shown in Fig. 1? Currently a previous study from the group is cited; however, details of the previous structural elucidation are lacking. AlphaFold3 is powerful but imperfect. Additional context would be helpful so that readers don’t need to refer to the previous study to understand the current one. Also, more information in-text about the biochemistry/structure of the c-di-GMP binding site would be helpful because it is a central player in the story. Likewise, additional rationale for the engineered HrcN mutations could be included in the text. Can the authors provide additional clarification?

**Author Response:** We have provided additional details about the predicted structure and location of the HrcN binding site, and the rationale for targeting the residues mutated in this study (line 115-120). Confidence intervals for our AlphaFold3 models are now included on line 366.

Line 130-131. In contrast to what is stated in the text, it appears as though the ATPase function of HcrN-G176A may be at least partially compromised. Consider that the Vmax for WT appears to be higher than that of the G176A mutant, and it is possible that 1.5 mM ATP – a concentration at which activity for WT is greater than the mutant – may be a physiologically relevant GTP concentration in live cells. Could the authors more carefully justify/consider their statements about activity? I worry that this result might confound interpretation.

**Author Response:** It is possible that the ATPase activity of our HrcN mutants is slightly compromised, and we now mention this as a possible confounding factor in the discussion (lines 244-247). However, we think it is unlikely that this change in ATPase activity fully explains the phenotypes we see for our mutants. Minamino and coworkers showed that very little ATPase activity was needed for full flagellar injectisome functionality (Minamino et al., 2014). Meanwhile, Erhardt and coworkers demonstrated that proton motive force can bypass the requirement for flagellar T3I ATPase activity entirely (Erhardt et al. 2014).

Minamino et al., 2014 – DOI: 10.1038/srep07579

Erhardt et al., 2014 – DOI: 10.1371/journal.pgen.1004800

Line 131-137 and Fig. 2. The crux of the paper seems to revolve around c-di-GMP binding to HrcN; however, the HcrN-G176A mutant – which is studied extensively in the manuscript – does not seem exhibit impaired c-di-GMP binding. This observation is difficult to understand in the narrative of the manuscript. Is it possible that the DRACALA assay is misleading here? The results in Fig. 2 C are not as clear as one might expect based on previous descriptions of the DRACALA assay (e.g., see Fig. 2 in the original PNAS paper from Vincent Lee’s lab describing the assay, <https://www.pnas.org/doi/10.1073/pnas.1018949108>). In fact, most of the results for competition assays appear to affect c-di-GMP binding relative to the solo fluorescent-c-di-GMP spot situated above them. G176A appears diminished in intensity relative to wild type. The proposed mechanism relies on a clear result here – could the authors please use a complementary technique to verify these data? The use of complimentary, validating techniques is a standard for protein-protein interactions to validate results. It is a big ask – but isothermal titration calorimetry or another biophysical protein-ligand binding technique might help to clarify the interpretation. As presented, the data here seem a bit murky.

**Author Response:** While our efforts to produce biophysical data for HrcN were unfortunately unsuccessful (see Figure R1, above) we were able to quantify our DRaCALA assay datasets and include this in Figure 2C. Our results suggest that cdG binding is unaffected in the G176A mutant, implying that this mutation may disconnect binding from downstream signal transduction in some way. We now discuss the possible mechanisms for cdG control of HrcN function in more detail on lines 247-254.

**Part III – Minor Issues: Editorial and Data Presentation Modifications**

Please use this section for editorial suggestions as well as relatively minor modifications of existing data that would enhance clarity.

**Reviewer #1:**

Figure 1D: Missing uninfected plant control, despite being mentioned in the text.

**Author Response:** We have remade this figure to include the missing plant control.

Absence of HrcN in Δ*hrcC* mutant needs explanation, does HrcN stability depend on the T3SS complex?

**Author Response:** This is indeed the case, HrcN stability depends on a stable assembly of the T3SS. With *hrcC* deleted, the T3SS lacks a proper basal body and so does not assemble, therefore HrcN is absent. We have added a sentence to clarify this in the text (line 130-132).

Band intensity in G176A appears weaker, possible instability or reduced expression? Adding a loading control (e.g., EF-Tu or RNApol) for normalization.

**Author Response:** Please see the response to Reviewer #2 in the Major Issues section, above.

Figure 2: Include a catalytically dead mutant as a negative control for ATPase activity.
DRaCALA data should be quantified. Fluorescence images alone are insufficient to assess subtle changes. In Fig. 2C, unlabeled cdG only partially competes for G176A, this warrants quantification and further interpretation.

**Author Response:** As stated above, we have extensively revised our biochemical analysis. As part of this, we have added ATPase data for an HrcN P142Q mutant that lacks functional ATPase activity to Figure 2A. We have also quantified our DRaCALA data, and present this in Figure 2C.

Figure 3: The water control (described in line 151) is not shown in the figure.
Figures S2 and S3 relate directly to Fig. 3 and should be merged for clarity.
The conclusion that cdG levels affect symptom development should address why both high and low cdG restore virulence.

**Author Response:** We have remade Figure 3 to include the water control leaves as requested. With respect to Figures S2 and S3, S2 relates to the two cdG manipulation experiments, while S3 provides supplementary material for immunocompromised plant infections. As these two figures relate to distinct lines of enquiry and a combined figure would be both large and complex, after careful consideration we would like to keep these as they are. We discuss possible reasons why we see virulence recovery with both high and low cdG levels on lines 255-262.

Figure 4: In Fig. 4B, labels are inconsistent, WT and G176A are mentioned instead of G311A.

**Author Response:** This was an error during figure preparation - 4B should of course refer to G311A. We have amended the label and apologise for the confusion.

Authors claim 5 effectors increased and 4 decreased in G311A, but only one each show statistical significance.

**Author Response:** This is a fair point. We have removed the line (previously line 171) talking about increased and decreased effector secretion in the G176A and G311A screening experiments and direct the reader to the more robust analysis in Fig 4C and 4D.

Δ*hrcN* control is mentioned but not shown in 4A or 4B.

**Author Response:** The cAMP generation values for our Δ*hrcN* controls in the experiments depicted in 4A and B were consistently very low for all effectors, in line with the values seen in 4C and 4D. For clarity, we therefore decided to omit these data from Figures 4A and B. We will include the raw and processed data for these samples in the supplementary datasets for the paper.

Effector order in panels A and B should be identical to facilitate comparison.

**Author Response:** We think the data in 4A and 4B is more visually understandable as a complete set, when presented from low to high. Therefore, we would like to retain the current order of the effectors in these figures, unless the reviewer strongly objects.

Legend is under-detailed: specify time post-infection, n values, statistical tests, and normalization methods.

**Author Response:** We have updated the legend to include these missing details.

Discrepancy between HopAF1 in Fig. 4A (non-significant) and Fig. 4C (significant) needs clarification.

**Author Response:** The datasets in Figures 4A and B are based on fewer samples than those in 4C and 4D, with a higher degree of error. This is now noted in the text on line 192-193 and was the reason we repeated these experiments with additional samples in 4C.

Why was HopAF1 not tested in 4D, despite its altered secretion profile in other panels?

**Author Response:** The Cya translocation assays in Fig 4 were both expensive and highly labour intensive. For these reasons we were forced to restrict our analyses in Fig 4C and D to a few exemplar effectors. We chose to follow HopAA1-2, with HopH1 as a control, over a time course to see whether compromised secretion levels recover over time (they don’t). We recognize that the secretion profile seen for HopAA1-2 may not be entirely the same for other compromised effectors and have added text on line 236-239 to discuss the time course results for HopAA1-2.

Figure 5: In 5A, HopH1 overexpression is phenotypic but not deleted in 5B why?

**Author Response:** HopH1 was chosen as a control because it is an effector whose *in-planta* role has been studied (Wei et al. 2007) but whose secretion, measured by ELISA assays, was unaffected by HrcN mutation. Furthermore, *hopH1* overexpression (overriding the HrcN G176A mutation) produced little/no effect on the overall virulence phenotype (Figure 5A), unlike the three other effectors tested. Consequently, we ruled out HopH1 as playing a role in the ‘asymptomatic’ phenotypes associated with our HrcN mutant. We have tweaked the text on lines 196-197 and 212 to clarify this.

C.-F. Wei, B. H. Kvitko, et al. *The Plant Journal* (2007) Vol. 51 Issue 1 Pages 32-46

**Reviewer #2:** -Also related to individual data used for the figures, all graphs show individual data as well as mean and variation. Such an increasingly used practice provides clarity and granularity to the data, and it is important as it helps to interpret results while contributing to avoid reproducibility issues.

-Complementation assays from Tn7 expression are very nice! Assays using plant defense mutants, as well as those including effector mutants are also appreciated.

**Author Response:** We thank the reviewer for their positive comments!

**Reviewer #3:** Overall, the manuscript is short and well written. However, there are a large number of abbreviations (e.g., Pto, T3I, T3E, etc.), and I wonder if some of the abbreviations might be removed. I leave this to the discretion of the authors.

**Author Response:** We have carefully reviewed the abbreviations throughout the paper. The four main ones used are *Pto*, T3I, T3E, and cdG. As all four are used extensively throughout the manuscript, we would like to keep these in place. A couple of abbreviations used less often (e.g. DGC) have been removed.

What is WspR19?

**Author Response:** WspR19 is an autoactive cdG synthase from *P. fluorescens* SBW25 that increases the concentration of cdG in the cell to well over 100 times the levels observed in WT. It is commonly used to manipulate cdG levels in different bacterial species. Our description of this protein has been expanded on line 169.

Fig. 1, panel D. I respect the truth in advertising; however, is there a possibility to increase specificity of the antiserum by using an adsorption protocol (thereby eliminating some of the extraneous bands)?

**Author Response:** This would certainly be possible. However, given the significant additional work including an adsorption step would involve, and that we have identified the most prominent non-specific band in our HrcN Western by mass spectroscopy, we would like to keep Figure 1D as is.

The fonts in Fig. S4 need to be adjusted – some are so large that they appear to overlap, others are so small that they are nearly illegible. Could the authors use a consistent, easily legible font size?

**Author Response:** We have revised the fonts in Fig S4 for consistency, as requested.
